# The association between sPD-1 levels versus liver biochemistry and viral markers in chronic hepatitis B patients: a comparative study of different sPD-1 assays

**DOI:** 10.1186/s12985-022-01777-3

**Published:** 2022-03-31

**Authors:** Wen-Juei Jeng, Chien-Hung Chen, Yi-Wen Wang, Mei-Hung Pan, Chia-Wei Lin, Chun-Yen Lin, Hwai-I Yang

**Affiliations:** 1 Department of Gastroenterology and Hepatology, Chang Gung Memorial Hospital, Linkou Branch, No. 5, Fuxing Street, Guishan Dist., Taoyuan City 333, Taiwan, ROC; 2 Department of Gastroenterology and Hepatology, Chang Gung Memorial Hospital, Kaohsiung Branch, No 123, Ta-Pei Road, Niao Sung Hsiang, Kaohsiung City, Taiwan; 3 Chang Gung University College of Medicine, Taoyuan City, Taiwan; 4 Genomics Research Center, Academia Sinica, 128 Academia Road, Section 2, Nankang, Taipei, 115, Taiwan; 5 Institute of Clinical Medicine, National Yang-Ming University, Taipei, Taiwan; 6 Graduate Institute of Medicine, College of Medicine, Kaohsiung Medical University, Kaohsiung, Taiwan

**Keywords:** Immune markers, PD-1, Assay, Hepatitis B virus

## Abstract

**Background:**

Soluble programmed death-1 (sPD-1) is a novel immune markers and possibly predictive of chronic hepatitis B (CHB) outcome. However, results were inconsistent by different ELISA kits. This study aims to compare the characteristics and correlations with other markers for sPD-1 measured by MyBioSource (MB) and R&D (RD) kits.

**Methods:**

A total of 254 untreated CHB patients from three sites were assayed with sPD-1 by MB and RD kits at the same time. Spearman’s correlations between the kits, and those with viral markers and ALT levels were calculated. Multivariate linear regression analysis was applied for independent factors associated with the sPD-1 levels.

**Results:**

There’s no correlation between sPD-1 level using MB and RD assays. sPD-1 by MB correlated profoundly with HBsAg (r = 0.8311, *P* < 0.0001), HBV DNA (r = 0.3896, *P* < 0.0001), and ALT levels (r = 0.1604, *P* = 0.0105) while an opposite trend by RD kit (r = − 0.0644, *P* = 0.3109; r = 0.2554, *P* < 0.0001; r = 0.4417, *P* < 0.0001, respectively for the 3 markers). In the multivariate linear regression analysis, HBsAg and ALT levels was the major factor associated with sPD-1 levels by MB and RD, respectively.

**Conclusions:**

The characteristics and correlations with host/viral markers of sPD-1 by the two kits are different and leading to different associations on clinical outcomes of CHB.

**Supplementary Information:**

The online version contains supplementary material available at 10.1186/s12985-022-01777-3.

## Background

Chronic hepatitis B virus (HBV) infection is a major health issue for its adverse consequences of cirrhosis, hepatic decompensation and hepatocellular carcinoma (HCC). Biomarkers nowadays applied for clinical outcome prediction are mainly HBV related. Serum HBV viral load and hepatitis B surface antigen (HBsAg) quantification were correlated with HBV replication and commonly used for predictive of milestone transition such as hepatitis B e antigen (HBeAg) seroconversion [1, 2] and HBsAg seroclearance [1, 3–6] or of adverse events like cirrhosis [7, 8] and hepatocellular carcinoma [9–12]. The chronicity of HBV infection results from exhausted T cell response, especially HBV-specific T cell, toward viral clearance [13, 14]. Programmed cell death protein-1 (PD-1) plays an immunosuppressive role that regulates T cell activation via inhibitory PD-1/program death-ligand 1 (PD-L1) pathway leading to immune tolerance [15, 16]. The blockage of PD-1 may recover dysfunctional intrahepatic T cell and virus-specific B cells response [17–19]. However, limited serum immunological marker be available that could stand for host immune response against HBV. Soluble programmed cell death protein-1 (sPD-1) is a novel seromarker which may reflect certain host immune response. The correlation between sPD-1 and clinical outcome, such as HCC, in CHB patients has been reported in several studies [20–22]. Nevertheless, discrepant range of sPD-1 and association results were observed among different studies which may due to different assay kits applied [23].

## Patients and methods

### Aim of the study

This multi-center study aims to investigate the characteristics of two commonly used sPD-1 kits (R&D and MyBioSource) by performing a head-to-head comparison on sPD-1 values assayed by two kits and analyzing their correlations with known HBV viral markers in CHB patients.

### Study design and setting

The experiment was performed in two stages: First, 15 healthy individuals who are both seronegative for HBsAg and anti-HCV Ab; Second, untreated chronic hepatitis B patients from three independent cohorts using stored serum. The stored serums of REVEAL cohort were sampled in 1992 and stored in − 30 °C refrigerator while the other stored serums of Chang Gung Memorial Hospital cohort in Linkou and Kaohsiung branch were sampled in recent 6 years and stored in − 80 °C refrigerators. To minimize the batch effect, standard samples were provided along with both MB and R&D assay kits and each experiment had been conducted according to the manufacturer’s protocol for quality control. A specific standard curve was generated using the standard samples for each batch of experiment. The experiments for MB and R&D assays were carried out as close as possible to minimize variation in sample storage.

### Patients recruitment

The serum samples of 254 untreated CHB patients were assayed with both sPD-1 kits manufactured by R&D (RD) and MyBioSource (MB) at the same time in three different research centers including REVEAL cohort (N = 135), Chang Gung Memorial Hospital Linkou branch (CGMH-LB; N = 47) and Kaohsiung branch (CGMH-KB; N = 72). All these patients have been documented with HBsAg seropositive at least 6 months prior to the assay. Other variables including age, gender, cirrhotic status, HBeAg seropositivity, serum alanine aminotransferase (ALT), HBV DNA, HBsAg quantitation level (qHBsAg) and HBV genotype were also recorded. The cirrhosis was assessed by abdominal ultrasonography with consecutive report of coarse liver parenchyma, deranged vascular structure, with or without small liver size, and or splenomegaly [24]. The study was conducted adherent to the ethical principles and regulation by Declaration of Helsinki 2000, and protocols were approved by IRB committee of Chang Gung Memorial Hospital (201802090B0, 201700467A3) and Academia Sinica (AS-IRB01-18061). Written informed consent was obtained from all subjects recruited in this study.

### Laboratory methods

Serostatus of HBsAg and HBeAg, and serum ALT level were assayed by commercial kits in the REVEAL cohort [9, 25] and by routine automated techniques at central laboratory system in CGMH-LB and CGMH-KB as previously reported [26, 27]. Serum HBV DNA was assayed using Roche Cobas Amplicor HBV monitor test (Roche Diagnostics, COBAS® Taqman® HBV Indianapolis, IN; detection limit: 12 IU/mL). Serum HBsAg level was measured by Roche Elecsys HBsAg II Quant assay (Roche Diagnostics, Mannheim, Germany; lower detection limit: 0.05 IU/mL). The sPD-1 levels were assayed by Human PD-1 DuoSet ELISA kit (R&D Sytems, assay Range: 156–10,000 pg/mL) and Human Soluble Programmed Death-1 (SPD-1) ELISA kit (MyBioSource, assay range: 125–4000 pg/mL) according to the protocols provided by the manufacturers. Unlike commercial assay plates provided by MyBioSource, R&D’s sPD-1 ELISA assay requires plate preparation by diluting the capture antibody before operation, and then aspiring, washing and blocking each well of the microplate twice.

### Statistical analyses

Comparison of continuous variables was performed by ANOVA or Kruskal–Wallis test depending on severity of violations of normality while by Chi-square or Fisher’s exact test for categorized variables. When sPD-1 levels were used as continuous variables, values greater than upper or smaller than lower detection limits of specific kits were recoded as those limit values (i.e. 10,000 pg/mL and 156 pg/mL for RD system and 4000 pg/mL and 125 pg/mL for MB system). Correlations of logarithmic sPD-1 level between MB and RD, and with different biomarkers were performed by Spearman correlation. Multivariate linear regression analysis was used to investigate independent host/virus factors that were associated with the sPD-1 levels assayed using the two kit systems and to estimate the β coefficient and corresponding 95% confidence interval (CI). *P* values less than 0.05 were considered as significant. All statistical analyses were done by SAS 9.4 (SAS Institute, Cary, NC).

## Results

### Patients demographic features

Among these 254 untreated chronic hepatitis B patients, mean age was 48.9 ± 9.6 year-old, 63.4% was male, 72.9% and 27.1% infected with HBV genotype B and C, respectively, 7.5% was cirrhotic, 95.3% was HBeAg seronegative and all untreated (Table 1). Comparing characteristics among the three sites, individuals from community-based REVEAL cohort were younger, less cirrhotic, lower for serum ALT, AFP, HBV DNA and HBsAg level than those from the other two hospitals. The distribution of sPD-1 level was comparable among three sites by MyBioSource kit. However when using R&D kit, the level of sPD-1 in REVEAL was much lower than that of the other two hospitals (Table 1). The sPD-1 level was much lower by using R&D kit than that by MyBioSource kit (*P* < 0.0001) in three CHB cohorts, similar tendency was also observed in the 15 healthy individuals [R&D vs. MB, median (range) sPD-1 level: 814.8 (156–10,000) versus 1391.9 (125–4000) pg/mL, *P* = 0.161].

**Table 1:** Characteristics of study participants among three centers

Variables	Total (N = 254)	Center			*P* value
		REVEAL (N = 135)	CGMH-KB (N = 72)	CGMH-LB (N = 47)	
*Gender*					
Female	93 (36.6)	49 (36.3)	15 (20.8)	29 (61.7)	< 0.0001
Male	161 (63.4)	86 (63.7)	57 (79.2)	18 (38.3)	
Age, years					
≤ 40	60 (23.6)	40 (29.6)	9 (12.5)	11 (23.4)	0.0052
40–60	152 (59.8)	82 (60.8)	44 (61.1)	26 (55.3)	
≥ 60	42 (16.6)	13 (9.6)	19 (26.4)	10 (21.3)	
Mean ± SD	48.9 ± 9.6	46.3 ± 8.7	52.7 ± 10.0	50.6 ± 9.5	< 0.0001
Median (IQR)	49.0 (41.0–56.0)	45.0 (39.0–52.0)	54.5 (46.5–60.0)	52.0 (41.0–57.0)	< 0.0001
*HBV genotype*					
B	140 (72.9)	79 (73.1)	53 (75.7)	8 (57.1)	0.3598
C	52 (27.1)	29 (26.9)	17 (24.3)	6 (42.9)	
*Cirrhosis*					
No	235 (92.5)	131 (97.0)	58 (80.6)	46 (97.9)	< 0.0001
Yes	19 (7.5)	4 (3.0)	14 (19.4)	1 (2.1)	
*HBeAg*					
Negative	242 (95.3)	134 (99.3)	69 (95.8)	39 (83.0)	0.0001
Positive	12 (4.7)	1 (0.7)	3 (4.2)	8 (17.0)	
*HBsAg, IU/mL*					
≤ 100	76 (30.4)	39 (28.9)	20 (28.2)	17 (38.6)	0.1291
100–1000	89 (35.6)	44 (32.6)	33 (46.5)	12 (27.3)	
≥ 1000	85 (34.0)	52 (38.5)	18 (25.3)	15 (34.1)	
Median (IQR), log_10_ IU/mL	2.6 (1.9–3.1)	2.7 (1.9–3.2)	2.4 (1.9–3.1)	2.6 (1.7–3.1)	0.5019
*HBV DNA, copies/mL*					
≤ 10^4^	127 (50.8)	81 (60.0)	28 (38.9)	18 (41.9)	< 0.0001
10^4^–10^5^	48 (19.2)	31 (23.0)	9 (12.5)	8 (18.6)	
≥ 10^5^	75 (30.0)	23 (17.0)	35 (48.6)	17 (39.5)	
Median (IQR), log_10_ copies/mL	3.9 (2.9–5.3)	3.8 (2.9–4.6)	4.8 (3.4–6.2)	4.2 (2.6–6.3)	0.0023
*ALT, U/L*					
≤ 15	96 (37.8)	86 (63.7)	4 (5.6)	6 (12.8)	< 0.0001
15–40	95 (37.4)	45 (33.3)	25 (34.7)	25 (53.2)	
≥ 40	63 (24.8)	4 (3.0)	43 (59.7)	16 (34.0)	
Median (IQR)	19.0 (10.0–39.0)	11.0 (7.0–19.0)	55.0 (28.5–170.0)	22.0 (18.0–55.0)	< 0.0001
*sPD-1 by MyBioSource, pg/mL*					
≤ 125	7 (2.8)	2 (1.5)	5 (6.9)	0 (0.0)	0.2079
125–4000	103 (40.5)	56 (41.5)	29 (40.3)	18 (38.3)	
≥ 4000	144 (56.7)	77 (57.0)	38 (52.8)	29 (61.7)	
Mean ± SD, log_10_ pg/mL	3.4 ± 0.4	3.3 ± 0.4	3.4 ± 0.4	3.5 ± 0.2	0.1425
Median (IQR), log_10_ pg/mL	3.6 (3.3–3.6)	3.6 (3.2–3.6)	3.6 (3.4–3.6)	3.6 (3.4–3.6)	0.5470
Mean ± SD, log_10_ pg/mL^a^	3.9 ± 1.0	3.9 ± 0.9	4.1 ± 1.1	N/A	0.2246
Median (IQR), log_10_ pg/mL^a^	4.0 (3.2–4.6)	4.0 (3.2–4.6)	3.9 (3.4–4.7)	N/A	0.4013
*sPD-1 by R&D, pg/mL*					
≤ 156	140 (55.1)	103 (76.3)	20 (27.8)	17 (36.2)	< 0.0001
156–10^4^	98 (38.6)	32 (23.7)	40 (55.5)	26 (55.3)	
≥ 10^4^	16 (6.3)	0 (0.0)	12 (16.7)	4 (8.5)	
Mean ± SD, log_10_ pg/mL	2.5 ± 0.6	2.3 ± 0.3	2.9 ± 0.7	2.6 ± 0.6	< 0.0001
Median (IQR), log_10_ pg/mL	2.2 (2.2–2.7)	2.2 (2.2–2.2)	2.8 (2.2–3.6)	2.3 (2.2–2.8)	< 0.0001
Mean ± SD, log_10_ pg/mL^a^	2.6 ± 0.6	2.3 ± 0.3	3.0 ± 0.8	N/A	< 0.0001
Median (IQR), log_10_ pg/mL^a^	2.2 (2.2–2.7)	2.2 (2.2–2.2)	2.8 (2.2–3.6)	N/A	< 0.0001

Values are expressed as number (percentage). HBV DNA and HBsAg data at sPD-1 assessment was available in 250 patients. HBV genotype was not available in 32 patients and failed genotyped in 30 patients

*ALT* alanine aminotransferase, *CGMH-LB* Chang Gung Memorial Hospital, Linkou Branch, *CGMH-KB* Chang Gung Memorial Hospital, Kaohsiung Branch, *HBV* hepatitis B virus, *HBeAg* hepatitis B e antigen, *HBsAg* hepatitis B surface antigen, *IQR* interquartile range, *SD* standard deviation, *sPD-1* soluble programmed cell death protein 1

^a^Serial dilution method for sPD-1 level above upper limit of assay, done in REVEAL and CGMH-KB only

### Comparison of sPD-1 levels between kits and known biomarkers

**Fig. 1:**
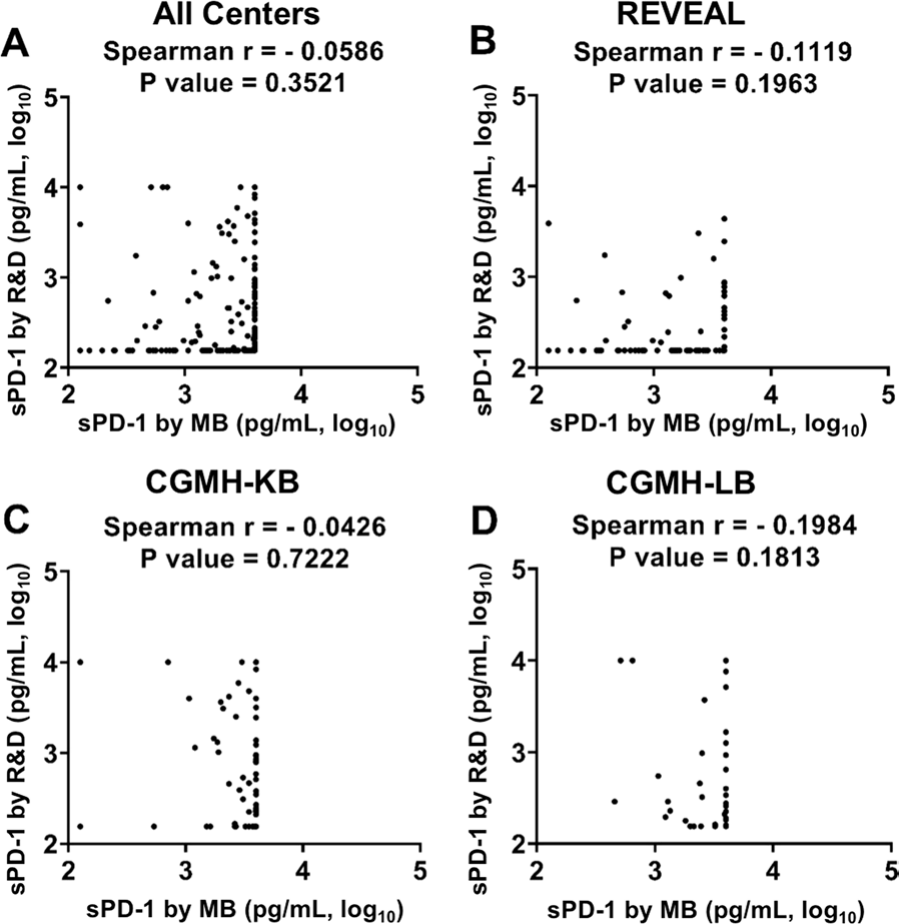
Correlations between serum sPD-1 levels measured by MyBioSource and R&D kits. Samples are from **A** all centers (N = 254); **B** REVEAL cohort (N = 135); **C** Chang Gung Memorial Hospital Kaohsiung Branch (CGMH-KB; N = 72) and **D** Chang Gung Memorial Hospital Linkou Branch (CGMH-LB; N = 47)

**Fig. 2:**
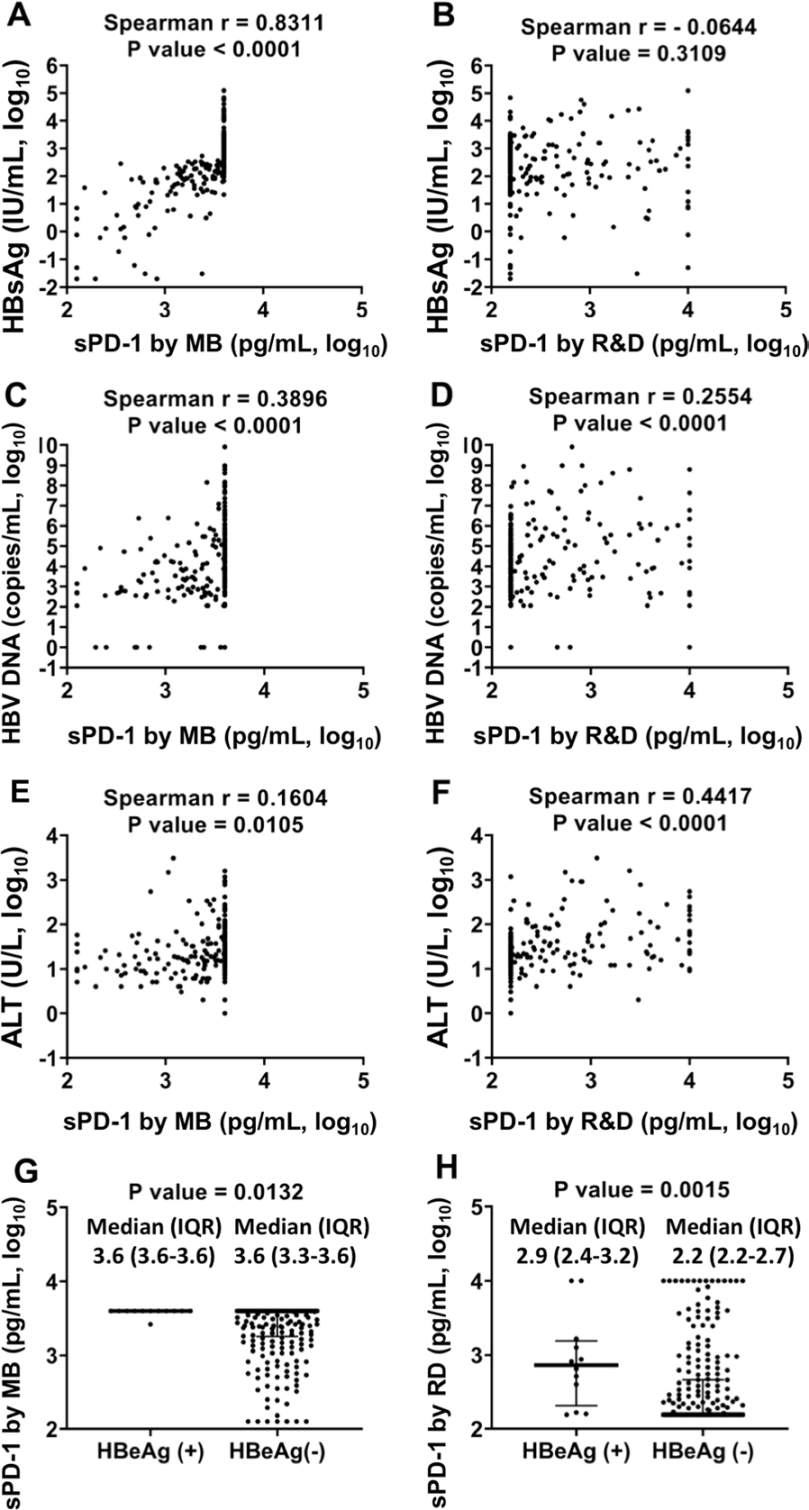
Association between serum sPD-1 levels measured by MyBioSource (MB) and R&D (RD) kits and HBV related markers including levels of hepatitis B surface antigen (HBsAg), hepatitis B virus DNA (HBV DNA), and alanine aminotransferase (ALT), and hepatitis B e antigen (HBeAg) serostatus in all centers. Correlations of serum sPD-1 levels measured by **A** MB with HBsAg levels; **B** RD with HBsAg levels; **C** MB with HBV **DNA** levels; **D** RD with HBV DNA levels; **E** MB with ALT levels; **F** RD with ALT levels; **G** MB with HBeAg serostatus; **H** RD with HBeAg serostatus

There is no significant correlation between serum sPD-1 levels measured by MB and RD, either merging all data together (Fig. 1A) or separating by center (Fig. 1B–D), nor even after dilution method (Additional file 1: Figure S4A–C). When examining the correlations of sPD-1 with viral markers, sPD-1 levels from MB were positively correlated with serum HBsAg levels (r = 0.8311, *P* < 0.0001; Fig. 2A; after dilution: r = 0.9219, *P* < 0.0001; Additional file 1: Figure S5A) while no significant correlation between sPD-1 levels by RD and HBsAg levels (r = − 0.0644, *P* = 0.3109; Fig. 2B; after dilution: r = 0.0581, *P* = 0.4065; Additional file 1: Figure S5B). Both sPD-1 values correlated with HBV DNA levels but was stronger in that from MB (r = 0.3896, *P* < 0.0001; Fig. 2C; after dilution: r = 0.4027, *P* < 0.0001; Additional file 1: Figure S5C) than that from RD (r = 0.2554, *P* < 0.0001; Fig. 2D; after dilution: r = 0.2593, *P* = 0.0002; Additional file 1: Figure S5D). Among both kits, sPD-1 levels were higher in HBeAg seropositive patients than that in HBeAg seronegatives (Fig. 2G, H, Additional file 1: Figure S5G, H). Nevertheless, the correlation between serum sPD-1 and ALT levels was stronger in that from RD (r = 0.4417, *P* < 0.0001; Fig. 2F; after dilution: r = 0.4034, *P* < 0.0001; Additional file 1: Figure S5F) than that from MB (r = 0.1604, *P* = 0.0105; Fig. 2E; after dilution: r = 0.2310, *P* = 0.0008; Additional file 1: Figure S5E). When separating by centers, the correlations between sPD-1 levels and levels of HBsAg and HBV DNA were consistence with the results of combining all centers (Additional file 1: Figures S1 and S2). However, the correlations between sPD-1 levels by MB and ALT was most significant in the REVEAL (r = 0.2266, *P* = 0.0082), while the correlations between sPD-1 levels by RD and ALT was most significant in the CGMH-LB (r = 0.6109, *P* < 0.0001; Additional file 1: Figure S3). Comparing the sPD-1 level between patients with (N = 19) and without (N = 235) cirrhosis, the median level was numerically higher in cirrhotic patients by RD assay (390 vs. 156 pg/mL, *P* = 0.767) while comparable by MB kit (3888 vs. 4000 pg/mL, *P* = 0.775). In the 15 healthy individuals, there’s no correlation between MB and R&D assays as well (r = 0.288, *P* = 0.298).

### Regression analysis for factors associated with sPD-1 levels

As many host and viral factors may affect sPD-1 level simultaneously, we used regression analysis to investigate independent factors that associated with the variation of sPD-1 levels (Table 2). In the univariate linear regression analysis, sPD-1 level by MB was associated with levels of HBsAg, HBV DNA and ALT, while SPD-1 by RD was associated with age and levels of HBV DNA and ALT. In the multivariate analysis, age [β coefficient (95% CI): 0.003 (0.0002–0.007), *P* = 0.0369] and HBsAg level [β coefficient (95% CI): 0.245 (0.217–0.273), *P* < 0.0001] were the two independent factors correlated with sPD-1 by MD; while gender [β coefficient (95% CI): − 0.152 (− 0.288 to − 0.016), *P* = 0.0288] and ALT [β coefficient (95% CI): 0.404 (0.267–0.540), *P* < 0.0001] were the independent factors associated with sPD-1 by RD.

**Table 2:** Linear regression analysis for factors associated with sPD-1 levels

Variables	sPD-1 by MyBioSource, log_10_ pg/mL				sPD-1 by R&D, log_10_ pg/mL			
	Univariate		Multivariate		Univariate		Multivariate	
	β coefficient (95% CI)	*P* value	β coefficient (95% CI)	*P* value	β coefficient (95% CI)	*P* value	β coefficient (95% CI)	*P* value
Gender (male vs. female)	0.001 (− 0.097–0.099)	0.9791	− 0.044 (− 0.107–0.020)	0.1795	− 0.046 (− 0.191–0.099)	0.5347	− 0.152 (− 0.288 − (− 0.016))	0.0288
Age, years	− 0.003 (− 0.008–0.002)	0.2344	0.003 (0.0002–0.007)	0.0369	0.009 (0.001–0.016)	0.0195	0.006 (− 0.0009 to 0.013)	0.0875
HBsAg, log_10_ IU/mL	0.237 (0.212–0.262)	< 0.0001	0.245 (0.217–0.273)	< 0.0001	− 0.024 (− 0.081–0.033)	0.4129	− 0.058 (− 0.118–0.002)	0.0591
HBV DNA, log_10_ copies/mL	0.070 (0.046–0.093)	< 0.0001	− 0.006 (− 0.027 to 0.014)	0.5507	0.065 (0.028–0.101)	0.0006	0.021 (− 0.023 to 0.064)	0.3542
ALT, log_10_ U/L	0.102 (0.018–0.186)	0.0176	0.025 (− 0.038–0.089)	0.4319	0.400 (0.285–0.515)	< 0.0001	0.404 (0.267–0.540)	< 0.0001

*ALT* alanine aminotransferase, *CGMH-LB* Chang Gung Memorial Hospital, Linkou Branch, *CGMH-KB* Chang Gung Memorial Hospital, Kaohsiung Branch, *CI* confidence interval, *HBV* hepatitis B virus, *HBeAg* hepatitis B e antigen, *HBsAg* hepatitis B surface antigen, *IQR* interquartile range, *SD* standard deviation, *sPD-1* soluble programmed cell death protein 1

## Discussion

Understanding the characteristic of one biomarker assayed by different commercially available kits is very important before investigating its clinical applications. In this study, we showed that the sPD-1 levels measured by MB and RD kits in the same serum samples were very different. The detection sensitivity for sPD-1 seem to be higher for kits manufactured by MB than that by RD in the same serum samples. More than half (55.1%) of the samples were under lower detection limit for sPD-1 assayed by RD while 97.2% were detectable assayed by MB, although the claimed lower detection limits were similar (156 pg/mL for RD vs. 125 pg/mL for MB). The difference was also reflected in the correlations with host and viral markers. sPD-1 by MB correlated profoundly with HBsAg levels, moderately with HBV DNA, rarely with ALT levels. Interesting, sPD-1 by RD showed an opposite trend. Since sPD-1 by R&D was positively correlated with serum HBV DNA (r = 0.2554, *P* < 0.0001) and ALT levels (r = 0.4417, *P* < 0.0001), the proportion of those with sPD-1 levels by R&D below the detection limit in REVEAL cohort was higher than two hospital-based cohorts (76.3% versus 27.8–36.2%). This is because 60% of the participants in the REVEAL cohort had HBV DNA lower than 10^4^ copies/mL and 63.7% of participants had ALT < 15 U/L. In order to exclude the possible confounding factor of sPD-1 protein stability by storinig condition, comparison experiments were done in CGMH-Linkou cohort on both stored serum samples (> 3 years) and newly collected samples (< 1 years), which were both stored in − 70 °C refrigerators, the same temperature as that in the other two cohorts. Linear correlations only existed between R&D’s sPD-1 level and ALT (*P* < 0.001) and HBV DNA (*P* = 0.07) but not between stored or fresh samples.

When all factors taken together in the multivariate linear regression analysis, HBsAg was the major factor associated with the sPD-1 levels measured with MB kits followed by age. Each log_10_ increment in HBsAg level contributed an increasing 0.245 pg/mL in sPD-1 by MB. On the contrary, each log_10_ increment in ALT level associated with an increasing 0.404 pg/mL in sPD-1 by R&D. The possible reason for this interesting phenomenon may be the different binding structure of sPD-1 in these two kits though we could not obtain relevant detail information from commercial website. From this point of view, the future utility or investigation for these two kits may be different and require larger sample studies for validation.

It has been shown that sustained exposure to antigens with high viral loads and excessive inhibitory signals in the liver microenvironment could lead to the exhaustion of HBV-specific T cells [28], which could be characterized by high PD-1 expression levels [29]. Thus, choosing the sPD-1 kit that demonstrated good correlations with HBsAg titer and HBV load in CHB patients seems to be a reasonable decision. In addition, the kit with better sensitivity in detecting sPD-1 would be more feasible for investigating its utility in predicting HBV milestone transition such as HBsAg loss or HBV DNA undetectability.

Our study suggests that the current published studies reporting the utility or prediction of sPD-1 on clinical outcomes shall be re-examined. Even with the same kit, the median level of sPD-1 by RD in our study was lower than that reported in another Taiwanese-based study (156 pg/ml vs. 293.3 pg/ml) [20], which did not mention the detection range but reporting levels lower than 156 pg/ml, the lower detection limit according to RD’s official manual. Further, more than half of our CHB individuals’ sPD-1 levels were below the detection limit when using RD. The correlation of sPD-1 by RD and HBsAg was poor in our study (r = − 0.0644, *P* = 0.3109) while that showed mild correlation in Zhou’s report (r = 0.2549, *P* < 0.0001) [22]. The sPD-1 levels assayed by the ELISA kit from Shanghai Enzyme-Linked Biotechnology Co. LtD or by the ELISA kit from Cloud-Clone Corp. Houston, Tx was also much higher than that reported in our current report [21, 30].

Though this is the first head-to-head comparison of sPD-1 by MB and RD, there are several limitations in our current study. First, the correlation of serum sPD-1 and the PD-1 level detected from PBMC or intrahepatic T cell remains unknown. Whether the serum level of sPD-1 could be correlated with or represent the PD-1 expression or exhaustion of T cells requires further study. Second, although both of the sPD-1 assays by MB and RD are commercialized products, we could hardly know the exact binding protein epitope sites of these two assays from their available official information. Judging from the obvious different characteristics of the two assays related to the clinical parameters of CHB patients, different binding epitope sites may be used by the different assays, but it needs to be confirmed by future mass spectrometry experiment. In the future, the two kits should be standardized using an universal standard substance of sPD-1 (if available), and the sample test values should be compared and adjusted to produce more accurate results. Further, we only compare the sPD-1 assayed by MB and RD. The difference between these two kits with the other brands is uncertain. Third, since we have only 12 HBeAg positive patients and only 3 of them are immune tolerant phase, it is difficult to demonstrate the difference of sPD-1 level across the clinical phases by these two kits. Forth, the utility of sPD-1 ELISA kit on clinical outcome prediction in CHB patients still require more investigations and validation.

## Conclusions

In conclusion, the sPD-1 level in CHB patients differs significantly by the ELISA kit assayed. sPD-1 from MyBioSource independently and majorly correlates with HBsAg levels while sPD-1 from R&D predominantly correlates with ALT. MB may be a better candidate platform for future sPD-1 research use as 97.2% CHB subjects could be detected with the kit. Whether the serum level of sPD-1 could stand for T cell exhaustion status or PD-1 expression requires further investigation. These issues regarding the discrepant characteristics between two different brands of sPD-1 assays shall be clarified prior to clinical application.

## Supplementary Information

**Additional file 1**. The association between serum sPD-1 (with dilution for absolute level) and viral markers and ALT levels.

## Data Availability

The data that support the findings of this study are available on request from the corresponding author. The data are not publicly available due to privacy or ethical restrictions.

## Abbreviations

ALT: Alanine aminotransferase
CHB: Chronic hepatitis B
HBeAg: Hepatitis B e antigen
HBsAg: Hepatitis B surface antigen
HBV: Hepatitis B virus
HCC: Hepatocellular carcinoma
HCV: Hepatitis C virus
MB: MyBioSource
QHBsAg: Quantitative hepatitis B surface antigen
PD-1: Programmed cell death protein 1
PD-L1: Program death-ligand 1
RD: R&D
sPD-1: Soluble programmed cell death protein 1
