# Supplementary material for: The association between sPD-1 levels versus liver biochemistry and viral markers in chronic hepatitis B patients: a comparative study of different sPD-1 assays
Source: Virol J. 2022 Mar 31;19:59. doi: 10.1186/s12985-022-01777-3 (PMC8973902; doi:10.1186/s12985-022-01777-3)
Supplement: Supplementary file 1 — Additional file 1. The association between serum sPD-1 (with dilution for absolute level) and viral markers and ALT levels. [file 12985_2022_1777_MOESM1_ESM.docx]

**Supplementary Figure Legends**

**Supplementary Figure 1. Correlations between serum sPD-1 and HBsAg levels stratified by sPD-1 kit used and centers.**


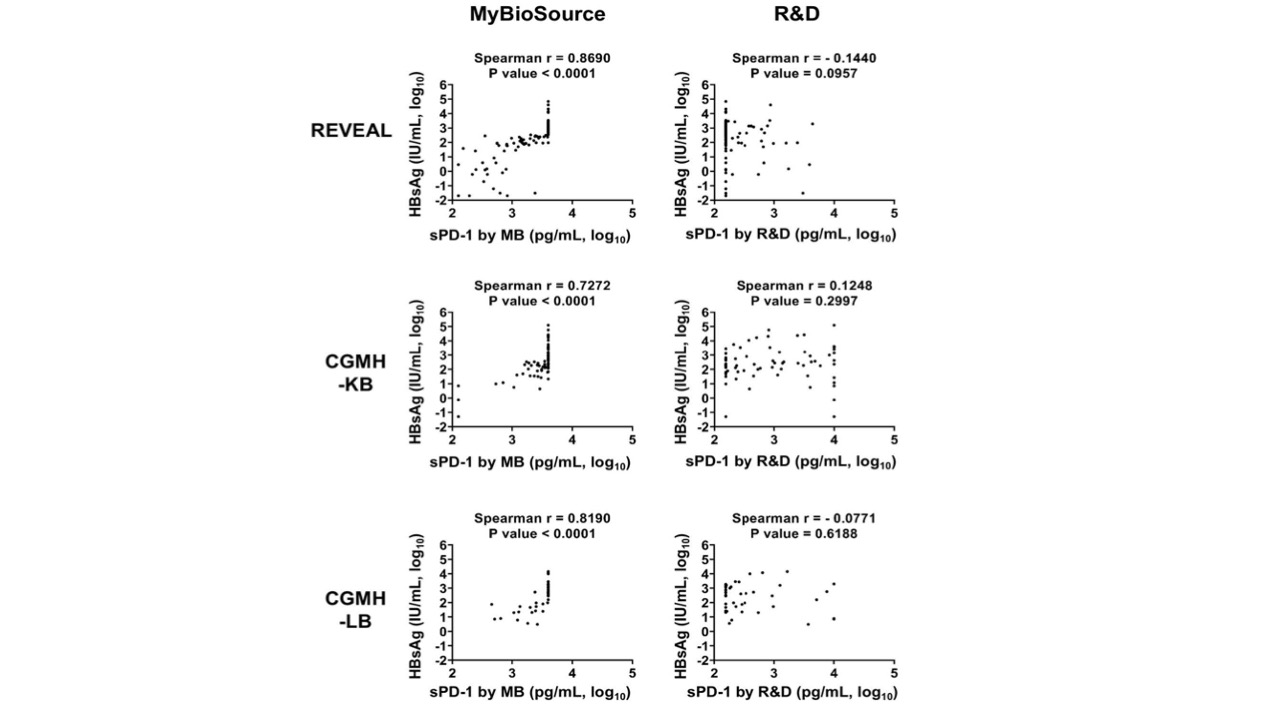


**Supplementary Figure 2. Correlations between serum sPD-1 and HBV DNA levels stratified by sPD-1 kit used and centers .**


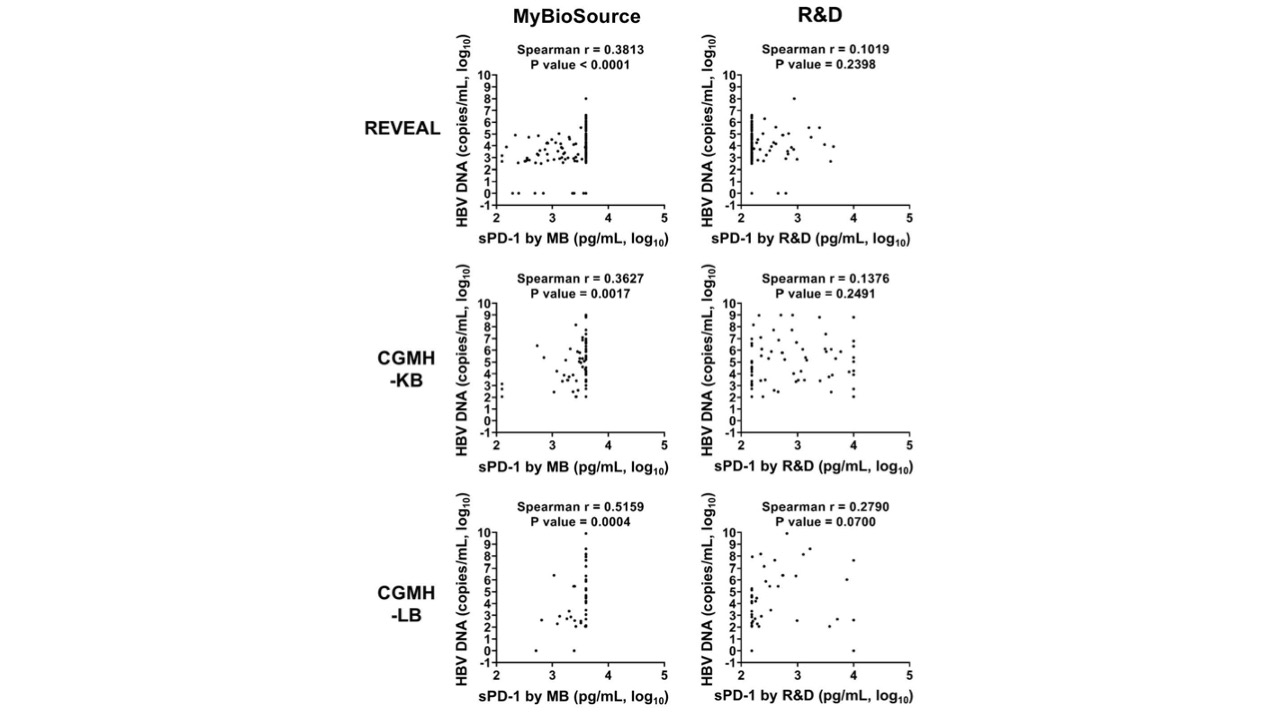


**Supplementary Figure 3. Correlations between serum sPD-1 and ALT levels stratified by sPD-1 kit used and centers.**

**
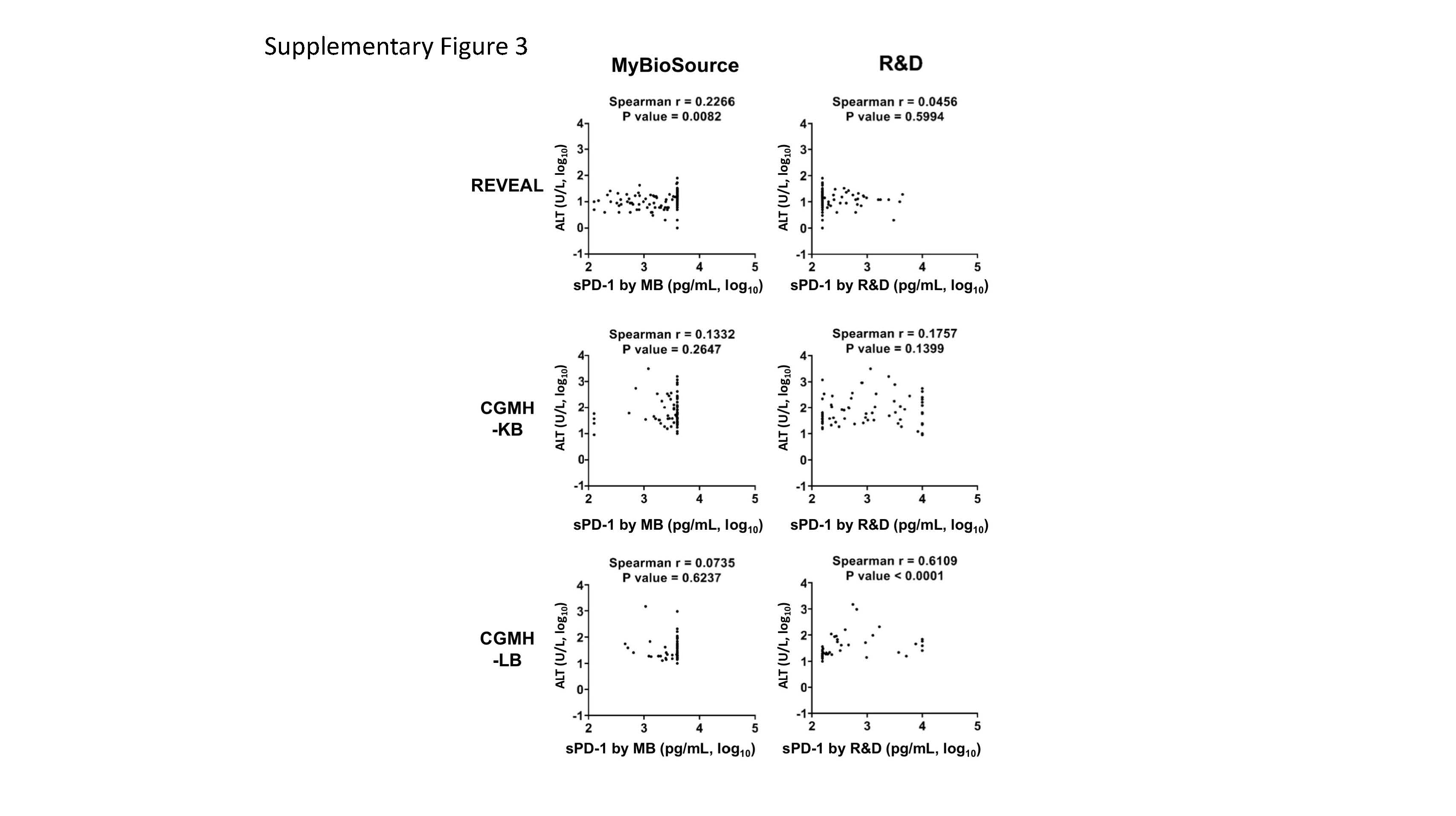
**

**Supplementary Figure 4. Correlations between serum sPD-1 by R&D and MB manufactures in different centers. Serial dilution was applied if exceed the upper limit of detection**

**
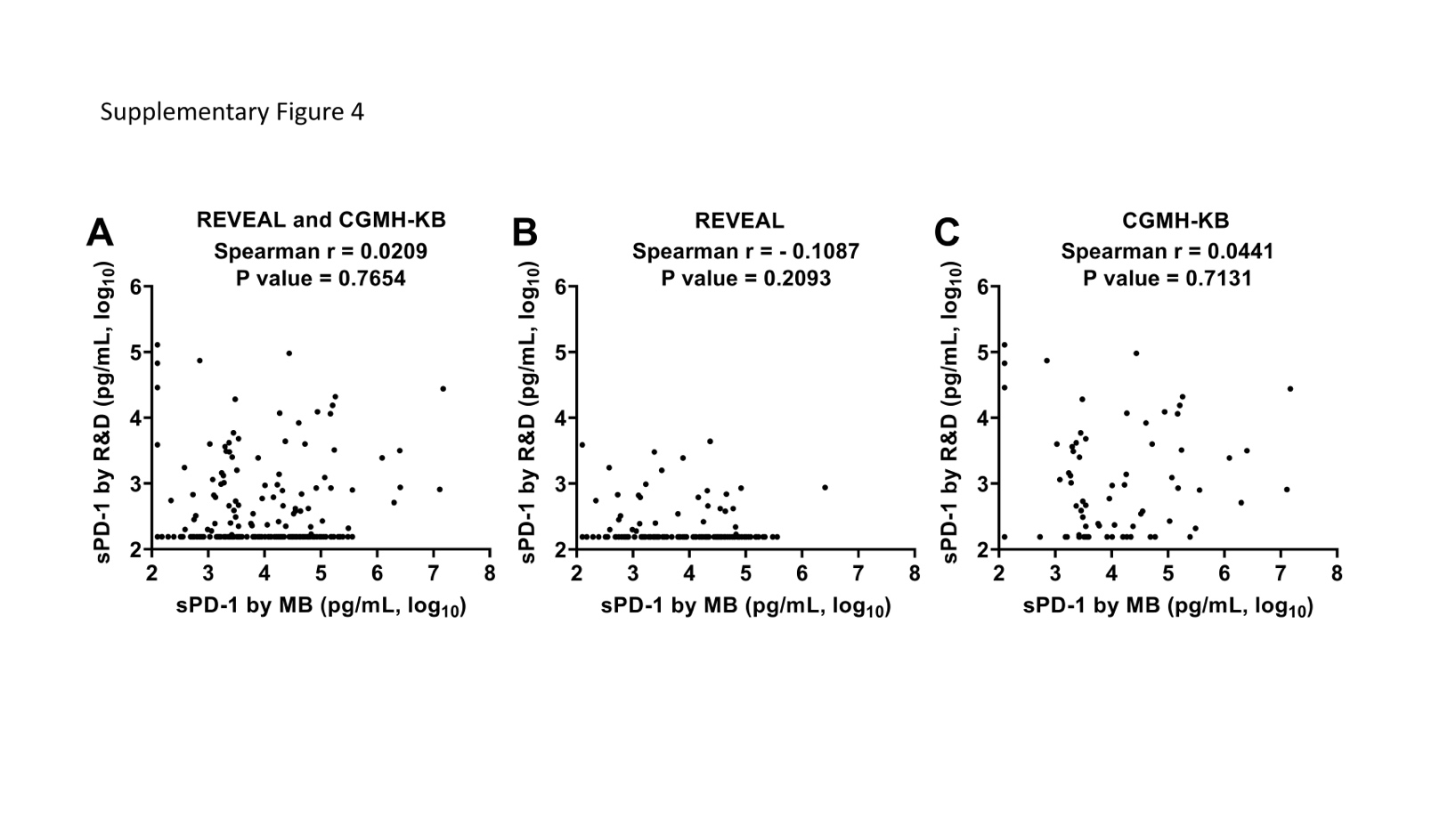
**

**Supplementary Figure 5. Association between serum sPD-1 levels measured by MyBioSource (MB) and R&D (RD) kits and HBV related markers including levels of hepatitis B surface antigen (HBsAg), hepatitis B virus DNA (HBV DNA), and alanine aminotransferase (ALT), and hepatitis B e antigen (HBeAg) serostatus in centers with serial dilution methods in patients whose sPD-1 level exceed the upper detection limit.** Correlations of serum sPD-1 levels measured by (A) MB with HBsAg levels; (B) RD with HBsAg levels; (C) MB with HBV DNA levels; (D) RD with HBV DNA levels; (E) MB with ALT levels; (F) RD with ALT levels; (G) MB with HBeAg serostatus; (H) RD with HBeAg serostatus.

**
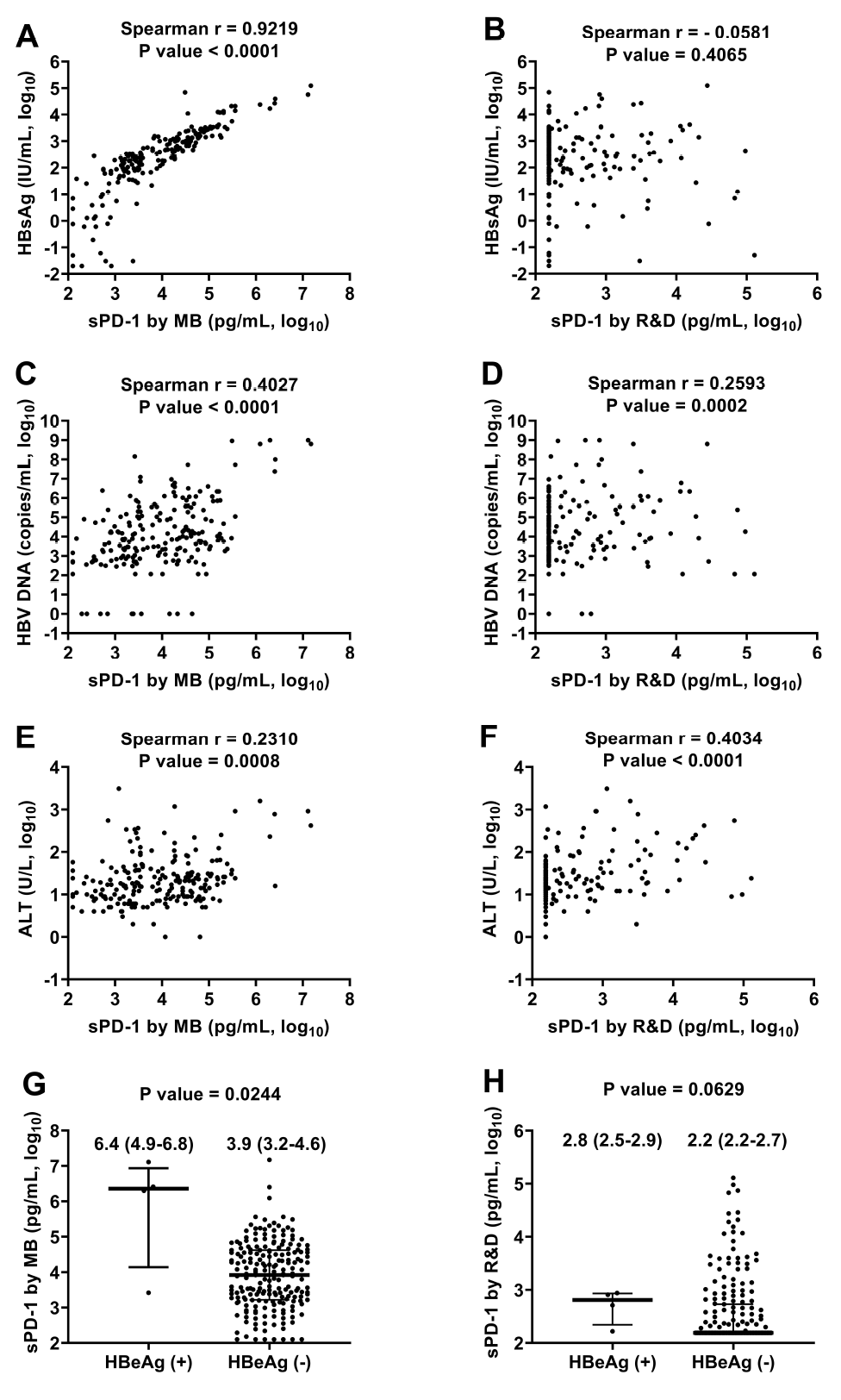
**
